# Heart-Puncture and Heart Suture as Therapeutic Procedures

**Published:** 1883-03

**Authors:** John B. Roberts


### HEART-PUNCTURE AND HEART-SUTURE AS THERAPEUTIC PROCEDURES. By JOHN B. ROBERTS, M.D.

(Read January 3, 1883.)

It is more than probable that, in a few years, puncture of the heart-wall (cardicentesis), with direct abstraction of blood by aspiration, will be recognized as the best treatment in cases of greatly dilated or much distended right heart, with intense pulmonary engorgement; and that incision of the pericardium with suture of the heart muscle will be accepted as proper in cardiac wounds. Hence these latest novelties in cardiac surgery deserve the attention of the Fellows of the College.

That punctures of the heart are comparatively harmless has been well known to many for some years. In 1872, Roger, while performing pericardicentesis on a child with pericardial effusion, thrust the needle into the right ventricle and withdrew about 6¼ Troy ounces (200 grams) of pure venous blood. The boy, who was aged five years, became pale, sweated, and had an imperceptible pulse. The withdrawal of the pericardial fluid, accomplished prior to the heart injury, was beneficial; and the cardiac puncture did no permanent mischief, for the patient recovered. Death occurred five months later from long existing dilatation and valvular disease of the heart (*Bull. de l'Académie de Médecine*, 1875, p. 1276).

In Hulke's case (*Trans. Clinical Society of London*, viii. p. 169), a woman with pleuro-pneumonia was supposed to have large pericardial effusion, and a trocar was introduced through the fourth left intercostal space. Nothing escaped except a drachm of venous blood, after which the patient seemed relieved of dyspnœa. She died four weeks later from a complication of diseases, and the autopsy revealed cardiac dilatation and valvular changes.

I have said elsewhere (*Paracentesis of the Pericardium*, 8vo., Philadelphia, 1880), in commenting upon this case: "The abstraction of blood seemed to relieve the distended heart much better than phlebotomy would have done, as was evinced by the diminution of threatening symptoms and the decrease of the area of dullness."

Cloquet, Bouchut, Legros, and Onimus have also observed the apparent innocuousness of wounds of the heart made by capillary trocars. Steiner found, ten years or more ago, that electro-puncture needles could be quite safely introduced into either ventricle, provided they were at once withdrawn (*Med. Times and*

*Gazette*, May, 1873, p. 492, from *Langenbeck's Archiv. für klin. Chirurgie*).

It has been considered less safe to puncture the auricles; but the interesting paper of Dr. Benj. F. Westbrook, just published in the *Medical Record* for December 23, 1882, seems to show that our fears are as unfounded as were those of our predecessors in regard to ventricular puncture. It is, in truth, to call attention to his case of harmless *intentional* cardicentesis and to his researches in the surgical anatomy of the operation, that I have been led to refer to the corroborative evidence of the cases mentioned above.

I have with much satisfaction, as have many others, done venesection at the bend of the arm for the temporary relief of the distressing symptoms of dilated heart, and for the dyspnœa due to the pulmonary engorgement of acute pneumonia. If, however, a few *drachms* of blood drawn directly from the heart give the relief that could only be afforded by taking a similar number of *ounces* from the veins of the arm, it seems proper to adopt the former measure. The subsequent circulatory depression from anæmia would undoubtedly be less than after the latter operation.

It is manifestly necessary, however, to determine that cardicentesis is innocuous before it can take the place of venesection. The above-mentioned cases and Dr. Westbrook's experience tend to show that such is the fact.

Dr. Westbrook believes that the proper place to perform the operation is in the third costal interspace close to the *right* edge of the sternum. This situation enables the operator to tap the right auricle without injuring the right internal mammary vessels, and with little danger of striking the tricuspid valve. My own preference would be to perforate the ventricle of the right heart by introducing the needle through the fourth interspace, about one and a half or two inches to the *left* of the median line of the sternum. Dr. Westbrook's opinion, however, is entitled to more deference than mine, because he has studied the subject with special reference to cardicentesis, while my special investigations have been limited to the consideration of pericardicentesis.

Further experimentation in heart-puncture for the relief of cardiac distension and pulmonary engorgement is requisite, but it is probable that it will soon become a well recognized surgical procedure in selected cases. Pericardicentesis has already taken that position, and there is no reason to believe that cardiac surgery will stop its march with the demonstration that the pericardium can be treated as the pleura.

In October, 1881, I read a paper before the Anatomical and Surgical Society of Brooklyn (The Surgery of the Pericardium; *Annals of Anatomy and Surgery*, December, 1881), in which I advised resection of costal cartilage and incision of the pericardium for removal of foreign bodies in the pericardial sac; and at the same time said: "The time may possibly come, when wounds of the heart itself will be treated by pericardial incision, to allow extraction of clots, and perhaps to suture the cardiac muscle."

It seems as if this time had now almost arrived, for Dr. Block has not only expressed a belief that death can be averted in many cases of heart-wounds by simple incision of the pericardium to allow escape or extraction of the clots which cause pressure and death, but has also undertaken to demonstrate by vivisectal experiments that suture of the heart is a simple operation, and requires but three or four minutes (*Amer. Jour. of the Med. Sciences*, Jan. 1883, p. 276; from *Journal de Méd. de Paris*, Oct. 28, 1882; from *Gaz. Méd. de Strassburg*, Oct. 18, 1882). He finds that opening of the right and left ventricles, and entire compression of the heart for the application of sutures, can be supported by rabbits for several minutes. During suturing, he seizes the apex of the heart and draws the organ forward until the traction prevents the escape of blood from the wound. Sutures are then introduced, or the orifice closed by ligation. Even if cardiac pulsation and the respiration stop during this mechanical interference with the heart's movements, death, he asserts, does not necessarily ensue.

These experiments are even more important than the researches spoken of in regard to heart-puncture. I regret that as yet I have not been able to consult Dr. Block's original memoir; but I hope at a future time to do so, and perhaps to be able to report some investigations of my own which I desire to make in the same direction.

